# Emotional Facial Expression Detection in the Peripheral Visual Field

**DOI:** 10.1371/journal.pone.0021584

**Published:** 2011-06-24

**Authors:** Dimitri J. Bayle, Benjamin Schoendorff, Marie-Anne Hénaff, Pierre Krolak-Salmon

**Affiliations:** 1 Lyon Neuroscience Research Center, INSERM U1028, CNRS UMR5292, Brain Dynamics and Cognition Team, Lyon, France; 2 Université Lyon 1, Lyon, France; 3 Diagnostic Imaging, Research Institute, Hospital for Sick Children, Toronto, Canada; 4 Hospices Civils de Lyon, Lyon, France; University of California, Berkeley, United States of America

## Abstract

**Background:**

In everyday life, signals of danger, such as aversive facial expressions, usually appear in the peripheral visual field. Although facial expression processing in central vision has been extensively studied, this processing in peripheral vision has been poorly studied.

**Methodology/Principal Findings:**

Using behavioral measures, we explored the human ability to detect fear and disgust vs. neutral expressions and compared it to the ability to discriminate between genders at eccentricities up to 40°. Responses were faster for the detection of emotion compared to gender. Emotion was detected from fearful faces up to 40° of eccentricity.

**Conclusions:**

Our results demonstrate the human ability to detect facial expressions presented in the far periphery up to 40° of eccentricity. The increasing advantage of emotion compared to gender processing with increasing eccentricity might reflect a major implication of the magnocellular visual pathway in facial expression processing. This advantage may suggest that emotion detection, relative to gender identification, is less impacted by visual acuity and within-face crowding in the periphery. These results are consistent with specific and automatic processing of danger-related information, which may drive attention to those messages and allow for a fast behavioral reaction.

## Introduction

The human visual system is constantly solicited by stimuli appearing randomly in all parts of the visual field. However, we do not behaviorally react to all stimuli. During the first steps of visual processing, salient stimuli are quickly detected, and a behavioral response is sometimes triggered. Facial expressions, especially fear, are considered salient stimuli [1]. The perception of emotional expressions is crucial for social communication and behavior [2], [3]. Identifying emotional expressions allows us to gather valuable information about others' moods and intentions and provides important clues as to the presence of environmental dangers. This is particularly true of fearful faces, which may convey information about imminent danger. For a social species, faces of congeners are naturally ubiquitous in the environment, often seen not just in the direct line of sight, but frequently appearing in the periphery of the visual field. It follows that there is an adaptive advantage to efficiently detect fear not only in the center but also in the peripheral visual field, allowing for a fast behavioral response to a nearby threat. Although a large body of research has been devoted to studying the perception of fear in the central visual field and some behavioral studies have investigated the perception of gender or identity in peripheral vision, relatively little work has concentrated on studying the perception of fear appearing in the peripheral visual field.

Previous behavioral studies have demonstrated declining performance in the peripheral visual field. The observed decline in identification performance with increasing eccentricity is different for an upright face, an inverted face or parts of faces, suggesting a predominance of configural processing during face identification [4]. More than part-based identification, configural processing is disturbed in peripheral vision by crowding between different parts of a face [5], [6]. This drop in peripheral vision performance can be compensated for by size and contrast scaling [7]. However, in real life, faces that appear in the peripheral visual field are not magnified. To our knowledge, only two studies have considered the effect of eccentricity on facial expression detection, but they either compared only two eccentricities in the close periphery [8] or used scaling factors to compensate for the loss of visual acuity [9]. Furthermore, in these two studies, performance in emotion identification was not assessed for the far periphery and was not compared with gender identification. Thus, the question of how and to what extent the visual system is, in ecological situations, able to detect the presence of emotional facial expressions in the periphery remains open.

Processing of facial expressions implicates specific pathways and dynamics distinct from those mobilized for processing other facial features, such as identity and gender [10], [11]. Structural face encoding mainly involves regions in the occipital and temporal lobes, including the fusiform face area [12] and the superior temporal gyrus [11]. This ventro-occipito-temporal visual pathway allows a fine-grained analysis of stimuli presented in the foveal region, which is more sensitive to high spatial frequencies than the peripheral retina. However, emotional expression can be detected from low spatial frequencies [13]. Moreover, low spatial frequencies seem to be involved in orienting attention toward fearful faces [13], suggesting a pre-attentional treatment of facial expressions [14] and allowing spatial attention to modulate the subsequent stages of facial expression processing [15].

Such pre-attentional processing could recruit a rapid visual pathway, mostly fed by magnocellular cells [16], [17] sensitive to low spatial frequencies and implicated in processing facial expressions [18], [19]. Projections to the amygdala through a subcortical extra-geniculate route involving the superior colliculus and the pulvinar have thus been postulated [20]. Indeed, the amygdala is centrally implicated in processing fear-related stimuli [21], and it has been suggested that such amygdalar processing might be pre-attentional [18]. However, it remains an open question whether such a fast processing pathway might be implicated in peripheral danger detection. As the peripheral visual field is very sensitive to low spatial frequencies, a peripheral stimulation would be particularly efficient to stimulate the rapid magnocellular visual pathway. Because danger often first appears in the peripheral visual field, it is conceivable that danger-signaling stimuli could be processed through a fast and automatic route. As facial expressions can convey danger-related signals, this would suggest a more efficient peripheral detection of facial expressions compared to other facial features.

The present study used a behavioral forced-choice paradigm aiming to explore the human ability to detect facial expressions in the extra-foveal visual field as a function of eccentricity. We chose to study two emotional facial expressions, fear and disgust, that both signal potential danger. As fear is more indicative of imminent danger than disgust, requiring a rapid behavioral response, we hypothesized that this expression would be better identified at far eccentricities than disgust. As a control, we used a gender discrimination task to test for the possibility of more efficient emotion detection in peripheral vision. Thus, we hypothesized that there would be better detection of emotion, especially fear, compared to gender in the peripheral visual field.

## Results

### Reaction times

**Figure 1:**
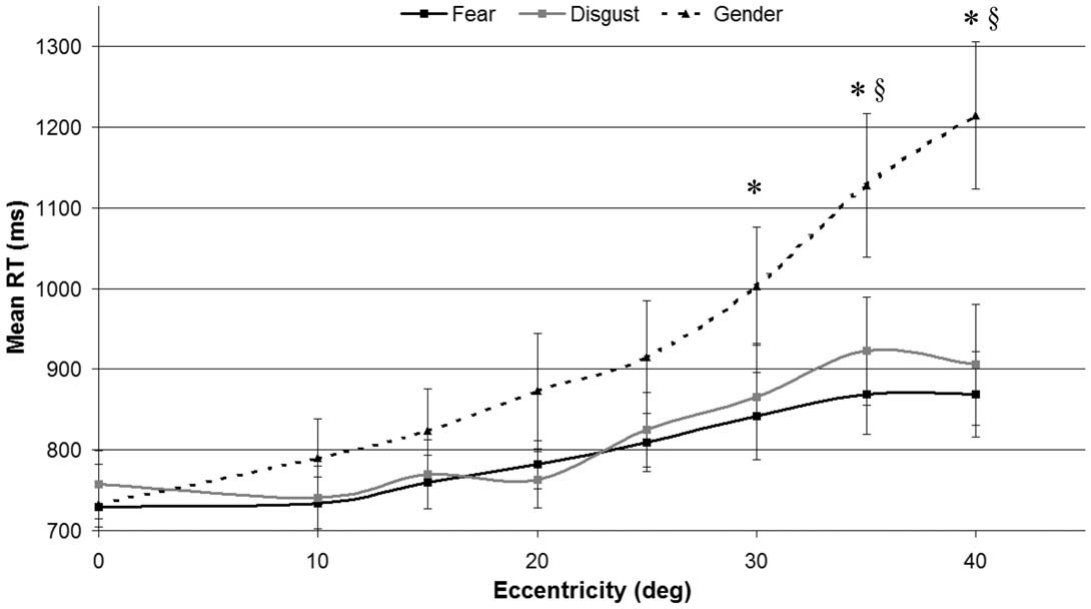
Reaction times as a function of eccentricity in the three discrimination tasks: * marks a significant difference between gender and fear discrimination reaction times, § a significant difference between gender and disgust discrimination reaction times. Vertical bars represent standard errors of the mean values.

The reaction times for correct responses for the 3 conditions and the 8 different eccentricities are shown in Figure 1. Mean reaction times were calculated for each subject and each condition, and a repeated-measure 2-factor ANOVA (eccentricity and condition) was conducted. There was a main effect of condition (*F(*2, 38*)*  = 9.04, *p*<.001, η_p_^2^ = .32), of eccentricity, (*F*(7, 133)  = 24.91, *p*<.001, η_p_^2^ = .57) and an interaction of condition by eccentricity (*F*(14, 166)  = 5.23, *p*<.001, η_p_^2^ = .22). The eccentricity effect corresponded to an increase in response time as a function of eccentricity. This effect, represented in Figure 1, was significant for fear (*F*(7, 133)  = 7.03, *p*<.001, η_p_^2^ = .27), disgust (*F*(7, 133)  = 5.59, *p*<.001, η_p_^2^ = .23), and gender discrimination (*F*(7, 133)  = 21.76, *p*<.001, η_p_^2^ = .53). *Post-hoc* Bonferroni corrected t-tests on the condition variable revealed no difference across eccentricities in the reaction times between the fear (M = 799, SD = 185) and disgust (M = 818, SD = 244) conditions, but the reaction time for gender discrimination (M = 935, SD = 334) was longer than that of fear (*p*<.001) and disgust (*p*<.001). *Post-hoc* analysis of each eccentricity showed that the difference in response time between the disgust and gender conditions was significant at 35° and 40° of eccentricity, whereas the difference between the fear and gender conditions was significant from 30° to 40° of eccentricity.

### Accuracy

**Figure 2:**
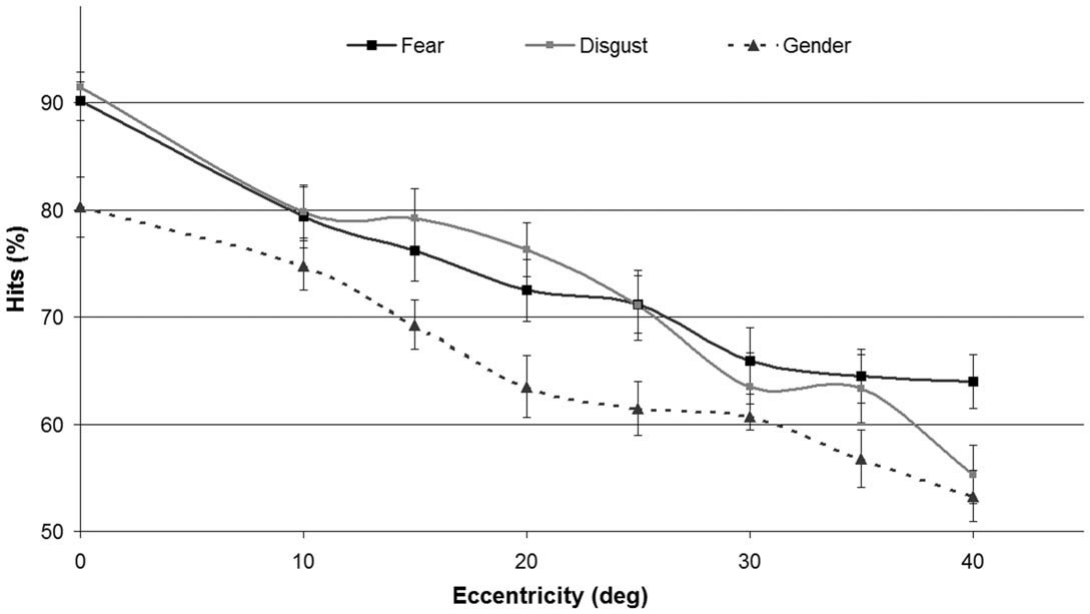
Percentage of correct responses in the three discrimination tasks as a function of eccentricity. Vertical bars represent standard errors of the mean values.

The percentages of correct responses were calculated for each subject in the 3 different conditions (gender discrimination, fear detection, and disgust detection) and for the 8 different spatial positions of target presentation (Figure 2).

We conducted a 3 (conditions) by 8 (eccentricities) analysis of variance (ANOVA) for repeated measures. This analysis revealed a main effect of condition (*F*(2, 38)  = 28.68, *p*<.001, η_p_^2^ = .60) and eccentricity (*F*(7, 133)  = 49.96, *p*<.001, η_p_^2^ = .72), but no interaction between the 2 factors (*p* = 0.24). The effect of eccentricity on performance was significant in the 3 conditions, with the more peripheral stimuli being less accurately discriminated. Accuracy performances were higher for emotion detection than for gender discrimination. Indeed, post-hoc Bonferroni t-tests conducted on the condition variables showed that accuracy for gender discrimination (M = 65.2, SD = 14.0) was lower than for fear (M = 73.0, SD = 14.4; *p*<.001) or disgust detection (M = 72.5, SD = 16.2; *p*<.001).

We tested at the highest eccentricities, where accuracy was the lowest, and when accuracy was above chance level (i.e., 50% in these tasks with 2 possible choices). A t-test revealed that, at 40° of eccentricity, the accuracy was not significantly different from the chance level for gender discrimination (*t*(19)  = 1.33, *p* = .2) or disgust detection (*t*(19)  = 1.93, *p* = .07), but remained above chance for fear detection (t(19)  = 5.59, *p*<.001).

## Discussion

The present study offers support for a visual system ability to detect emotional facial expressions at increasingly peripheral visual field eccentricities of up to 40°. Compared to gender, emotion was better detected in the peripheral visual field, and interestingly, the performance differential increased with increasing eccentricity. Moreover, the emotion detection advantage was higher for fearful faces than for disgusted faces, with detection accuracy remaining above chance level at 40° of eccentricity.

By presenting facial expressions in different spatial positions, the present study highlights an impressive ability of the visual system to detect emotions at very high eccentricities. Despite the short presentation time used, disgusted faces were recognized as emotional faces above chance level as far as 35°, and fear remained detectable at 40° of eccentricity. Facial expression is a particularly salient component, especially the expression of fear, which is usually indicative of threat-related stimuli. The ability to quickly detect potential threats in the environment and react appropriately is essential for survival. It would thus make sense that such a skill has been selected phylogenetically. This skill largely depends on visual function, yet in humans, visual resolution strongly decreases as a function of retinal eccentricity and is drastically reduced at 40° of eccentricity [22]. In spite of this, emotional facial expression detection remains effective in the far peripheral visual field, supporting our hypothesis that visual structures afferented by the peripheral retina, hitherto known for their motion detection properties, may also be able to process emotionally relevant and salient clues critical for social cognition.

The peripheral retina, contrary to the fovea, is mostly linked to the magnocellular visual system. Thus, facial expression processing in the peripheral visual field may implicate neural structures fed largely by magnocellular cells. This hypothesis has been proposed in previous studies suggesting an important role of low spatial frequency information in facial expression perception [13]. Because low spatial frequencies are mostly conveyed by the magnocellular system, this system could be preferentially involved in facial expression processing. Furthermore, the magnocellular pathway is adapted to detect stimuli salience, especially high-contrast information [23]. As salient high-contrast information coming from the eye region is different between neutral and emotionally expressive faces, this could explain the detection advantage observed for facial expressions [24]. Our study demonstrated effective processing of peripheral emotional information. This result provides behavioral support for the major involvement of the magnocellular pathway in facial expression processing in peripheral vision. This finding is in accordance with a previous neuroimaging study [13] but is in contrast with a behavioral study in which facial expression recognition was found to be more impaired by filtering low rather than high spatial frequencies [9]. This discrepancy may be explained by a difference in tasks. Explicit emotion recognition in the Goren and Wilson study could require fine analysis by the parvocellular system, while implicit recognition in the Vuilleumier et al. study or detection in the present study do not require such analysis and mainly involve magnocellular processing.

Our results provide clear evidence of a difference between gender and emotion processing in the peripheral visual field. The accuracy of detection performance was higher for emotion both in the central visual field and in peripheral vision, and reaction times were shorter for emotion detection in eccentricities greater than 30°. The absence of salient gender-specific features such as hair or beards in the stimuli might account for the lower accuracy performance in gender discrimination in foveal vision. However, the difference between response times in gender vs. emotion detection increases with eccentricity. Response times were not different between emotion detection and gender discrimination for central and low eccentricity presentations, and in the three tasks, the higher the eccentricity was, the slower the responses. However, the rate of slowing down from the center to 40° of eccentricity was almost 500 ms in the gender condition, whereas it was less than 150 ms in the emotion detection conditions. From 30° to 40° of eccentricity, subjects were significantly quicker in detecting emotion than discriminating gender. This demonstrates a qualitative difference in processing between gender and emotion, with emotional detection being less affected by increases in eccentricity.

Such a difference has already been demonstrated for central vision in a behavioral study [10] and is supported by neuroimaging studies [11]. In particular, it has been shown that, in contrast to facial expression perception, gender discrimination is mainly performed by the extraction of configural information from the face [25], requiring a fine analysis of facial features. This could explain how emotion detection performance is less affected by increases in eccentricity than gender discrimination performance. Indeed, the fine analysis required for gender processing is mainly performed by the parvocellular visual system, while, as suggested above, emotional facial information might implicate neuronal visual pathways sensitive to magnocellular information. As the magnocellular system is essentially afferented by the peripheral retina [26], the loss of visual acuity with increasing eccentricity is partially compensated for by the use of magnocellular information (i.e., low spatial frequencies and high contrast) for facial expression perception.

The drastic loss of efficiency for gender compared to emotion perception in the far periphery might also reflect a crowding effect, affecting gender perception in particular. Crowding reflects the fact that a target is less recognizable when presented with neighboring objects. This interference between objects has been described at length and particularly for letters. Crowding between the different parts of a face has been reported to affect its identification in peripheral vision by perturbing feature-based analysis [6]. Given that gender discrimination requires more feature-based analysis than emotional detection, this type of discrimination would be more impacted by crowding in the peripheral vision.

At the highest eccentricity, emotion was detected above chance level (64%) for fearful faces, while for disgusted faces, emotion detection was not significantly different from chance level (55%). Furthermore, shorter reaction times for emotion compared to gender discrimination were observed from 30° of eccentricity for fear, but only from 35° for disgust. This result might seem to contradict behavioral studies of centrally presented emotional faces that indicate that fearful expressions are detected more slowly and less accurately than other expressions, including disgust [27], and that, in go/no-go paradigms, response times to fearful faces are slower than those to neutral or happy faces [28]. It must be noted that in our experiment, we assessed the detection of emotional expressions, whereas the pre-cited studies assessed the identification of emotional expressions. Moreover, our results indicate that the advantage for emotion detection compared to gender discrimination is increased by peripheral presentation and that this advantage for emotion has a tendency to be larger for fear than for disgust. This could be explained by a preference of the magnocellular visual pathway for fearful expressions and would be coherent with the functional role of this pathway. As suggested by many authors, this pathway may be used for the rapid detection of salient threat-related stimuli [19], [29], [30]. Fearful faces, being more threat-related than disgusted faces, would more efficiently trigger a rapid brain reaction, particularly in the peripheral visual field. Consistent with this hypothesis, the rapid magnocellular visual pathways would be more involved in the detection of fear than other facial expressions [31]. However, despite an observed tendency, no significant differences were observed between disgust and fear in the present study. Further investigation will be necessary to demonstrate an advantage for fearful detection compared to the detection of other emotions in the peripheral visual field.

### Conclusion

The present study reveals that the visual system is able to detect the presence of facial expressions presented at very high eccentricities. Compared to gender perception, the emotion detection ability is less affected by the decrease in visual acuity and within-face crowding that comes with increasing eccentricity. At very high eccentricities, emotional expression detection is more efficient for fear than disgust, as fear is more threat-related than disgust. The effective detection of facial expression in the periphery could confer an adaptive advantage, as such danger-related stimuli require a fast and adapted behavioral response. As magnocellular processing is less affected by eccentricity and as the perception of fearful expressions requires low spatial frequency processing, fear detection is favored in peripheral vision. Our data lend support to the hypothesis that the magnocellular system is involved in the detection of facial expressions. However, further investigations will be necessary to determine which facial features in particular can be detected by such a system. As suggested by neuroimaging studies, facial expression perception could involve a rapid neural route to the amygdala, bypassing the occipital cortex [19], [30]. The present results are consistent with the involvement of such a rapid pathway that is sensitive to the magnocellular system [17] in danger-related stimuli processing. By demonstrating that peripheral vision is surprisingly competent in the processing of some facial features, we propose that much might be learned from further studies in the field combining behavioral measures with electrophysiological and neuroimaging studies.

## Methods

### Ethics statement

Each subject provided informed written consent. The study was conducted in accordance with the Declaration of Helsinki and was approved by the French ethics committee, Comité de protection des personnes SUD-EST IV, centre Leon Bérard.

### Participants

Twenty volunteers (10 men, 10 women) between the ages of 18 and 31 years (mean 23.55; sd 3.47), participated in the experiment. None had psychiatric or neurological disorders, and none were under pharmaceutical treatment at the time of testing. All volunteers were paid for their participation. Informed written consent was obtained.

### Stimuli and experimental setup

**Figure 3:**
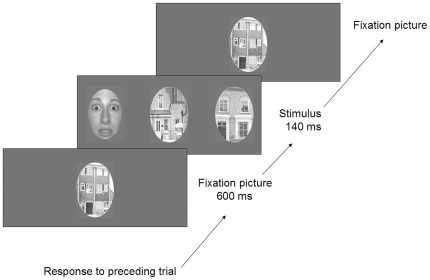
Description of a trial: A fixation stimulus (a house picture) centrally presented for 600 ms, is followed by the target stimulus presented for 140 ms. The fixation stimulus is then presented until the subject's response.

Sixty black and white photographs of faces of 20 individuals (10 males), each presenting 3 emotions (20 neutral, 20 fearful and 20 disgusted), and 10 black and white photographs of houses were used to construct the stimuli. Thirty-six faces were selected from the Nimstim set (http://www.macbrain.org/resources.htm), 15 were selected from the Ekman set [32], and 9 photographs were taken by ourselves and tested for emotion and gender in the 20 subjects. All photographs were brightness-adjusted, and all the presented faces had the same global (RMS) contrast [33]. Faces and houses were presented in ovals measuring 140×100 mm and subtending in the central position at a visual angle of 7.5° horizontally and 10.5° vertically when presented onto the screen. Each stimulus consisted of three horizontally aligned ovals, one presented centrally and two presented laterally and symmetrically at one of seven eccentricities: 10°, 15°, 20°, 25°, 30°, 35° or 40°. Eccentricities were measured from the center of the central oval to the center of the peripheral oval. One of the three ovals was a face, and the others were houses used as fillers (Figure 3). Each trial consisted of the presentation of the fixation picture (a picture of a house) at the center of the screen for 600 ms, followed by the presentation of the stimulus for 140 ms, immediately followed by the presentation of the fixation picture on the screen for up to 600 ms after the subject's response (Figure 3) The stimulus presentation time was kept below 150 ms to avoid any ocular saccade toward the target. To minimize memorization effects of stimuli presented centrally and so clearly identified, each face was first presented at the furthest degree of eccentricity (40°) and then gradually moved toward the center during the run through all successive eccentricities. This peculiar order of target presentation would induce a bias, with a lot of faces at high eccentricities at the beginning of the run and, conversely, a large number of faces at low eccentricities at the end. To avoid this bias, we used “filler” faces (same proportion of male/female and neutral/emotional faces as for the target stimuli) to provide an impression of randomization in stimuli position. These fillers were presented at semi-randomly chosen eccentricities during the run to ensure a pseudo-randomization of the eccentricity and position of the whole set of faces. The responses to these fillers faces were not entered in the analyses.

Stimuli were presented using *Presentation™* software on a large screen from a 2000 ANSI *Sony™ VPL-CX6* projector placed behind the subject. Responses and response times were recorded by a 2-button response.

### Experimental conditions and procedures

Participants were seated in a soundproofed room, facing the screen, with their chins resting on a chin rest and their eyes being horizontally aligned with the stimuli at a distance of 77 cm from the screen.

Participants were presented with 3 successive forced-choice tasks:

### One gender discrimination task

Instructions were to discriminate gender by a button press as accurately and fast as possible and to answer even in the absence of certainty. For each presented stimulus, subjects were asked to answer female or male by pressing the corresponding button responses. A total of 160 target stimuli (female/male  = 1) and 70 fillers were presented, corresponding to 20 target stimuli for each of the eight eccentricities of stimulation (including the center). Half of the target stimuli were neutral faces, and half were emotional faces. Half of these emotional faces expressed fear and half disgust.

### Two emotion detection tasks

Subjects were asked to detect, by a button press, the presence of an emotional expression as accurately and as quickly as possible even in the absence of certainty. They had to answer whether there was or was not an emotion in the presented face by pressing the corresponding button. As for the gender discrimination task, 160 target stimuli (20 per eccentricity) and 70 fillers were presented. For each eccentricity, half of the 20 presented targets were neutral, and half were expressive. During the run, all of the emotional faces expressed the same emotion, i.e., fear or disgust.

This task was run twice for each subject, once with fearful faces and once with disgusted faces. To ensure that there was no bias due to the run order, half of the participants started the emotion discrimination task with fear/neutral discrimination, while the other half started with disgust/neutral discrimination.

For all runs, participants were encouraged to keep their gaze on the fixation stimulus that remained projected on the center of the screen in between stimulus presentations and throughout each experimental block so as not to miss any of the stimuli appearing in the periphery. Assignment of the mouse response buttons was switched for a randomly chosen half of the participants.
